# The relationship between health literacy and health-related quality of life among school-aged children in regional China

**DOI:** 10.1186/s12955-021-01895-6

**Published:** 2021-11-25

**Authors:** Huifen Qiao, Xiaorong Wang, Zhenzhen Qin, Na Wang, Ning Zhang, Fei Xu

**Affiliations:** 1 Nanjing Brain Hospital Affiliated to Nanjing Medical University, 264, Guangzhou Road, 210029 Nanjing, China; 2 Department of Pediatric Intensive Care Unit, Children’s Hospital of Nanjing Medical University, Nanjing, China; 3 Nanjing Municipal Center for Disease Control and Prevention, 2, Zizhulin, Nanjing, 210003 China; 4 Department of Epidemiology, School of Public Health, Nanjing Medical University, Nanjing, China

**Keywords:** Health literacy, Health-related quality of life, CHU9D, Children and adolescents, Primary and high school students

## Abstract

**Objectives:**

To examine the association between health literacy (HL) and health-related quality of life (HRQoL) among primary and high school students in Nanjing, China.

**Methods:**

A cross-sectional study was conducted among randomly selected primary (graders 4–6), junior (graders 7–9) and senior (graders 10–12) high school students in 2018 in Nanjing Municipality of China. HRQoL, the outcome variable, was assessed with the validated Chinese version of Child Health Utility 9D (CHU9D) and used as continuous variable, while HL, our independent variable, was measured with the validated Chinese Students’ Health literacy Assessment Scale and treated as categorical variable (“adequate” or “inadequate”) in the analysis. Mixed-effects linear regression models were introduced to calculate mean difference and 95% confidence interval (CI) for examining the association between HL and HRQoL.

**Results:**

Totally, 4388 of 4498 students completed the survey. Among these responders, the mean score of CHU9D was 0.78 ± 0.17, and the proportion of participants with adequate HL was 85.8% (95% CI = 84.7%, 86.8%). After adjustment for potential confounders and class-level clustering effects, participants who had adequate HL were observed having, on average, an elevated HRQoL score of 0.08 (95% CI = 0.06, 0.11) units compared to their counterparts with inadequate HL. Such a positive HL-HRQoL association was also identified among each stratum of participants’ age, gender and residence.

**Conclusions:**

HL was positively associated with HRQoL score among primary and high school students in China. It has public health implications that HRQoL may be improved through school-based health literacy intervention among children and adolescents in China.

## Background

Health-related quality of life (HRQoL), a broad self-perception regarding physical and mental health conditions, is a comprehensive indicator usually used for both clinical and public health purposes to measure individual’s life quality [1–3]. HRQoL has been examined to be associated with some lifestyle and behaviors among children and adolescents in Western countries and China [4–11]. This implies that lifestyle and behavior intervention may be of help for improving HRQoL among children and adolescents. And, actually, HRQoL has been recently applied to assess the effectiveness of population-based lifestyle and behavior intervention campaigns for children and adolescents [12, 13].

Health literacy (HL) refers to the degree to which individuals have the capacity to obtain, understand, and use basic health information and services to inform health-related decisions and actions for themselves and others [14, 15]. Four domains, mainly including health-related knowledge, belief, skills and behaviors, are usually integrated into a scale to assess personal HL level among both adult and adolescent population worldwide [16–20]. HL was identified to be positively associated with HRQoL within adults [21] and school-aged children and adolescents [22–24]. Among these very few available studies on the relationship between HL and HRQoL among school students, participants were limited to those students with a narrow range of age-groups (11–13, 15–19 or 12–15 years old) and thus the separate results could not be extrapolated to general population of children and adolescents [22–24].

A better understanding of the relationship between HL and HRQoL among children and adolescents will be of help for HRQoL promotion through population-based health education and HL intervention programs. However, to date, there is no study available on the HL-HRQoL association from children and adolescents across wide age groups (e.g., including primary, junior high and senior high school students in a study) in a single study. China is the most populous country in terms of either overall population or school-aged children/adolescents in the world. Therefore, it is of particular public health importance to explore the association between HL and HRQoL among representative participants of children and adolescents in China. In this study, we hypothesized that HL was in positive relation to HRQoL among either primary or high school students in China.

## Methods

### Study design and aims

Data used in this study were from a large school-based survey, The Built Environment and Chronic Health Conditions: Children (BEACH-Children) Study [25], which was conducted among school-aged children and adolescents between May and June of 2018 in Nanjing, a typical mega-city in China. The BEACH-Children Study was developed as a cross-sectional survey with three main objectives to investigate: (1) the link between built environment and obesity and physical activity (PA); (2) the association of lifestyle and behaviors with HRQoL; and (3) HL prevalence and its associated characteristics. In the present study, we aimed to specifically examine the HL-HRQoL association among students from primary, junior high and senior high schools in regional China.

### Participants’ eligibility and selection

There were, in total, 8.3 million people registered in 12 administrative districts of Nanjing in 2018 [26]. Meanwhile, in the academic year of 2017–2018, approximately 667,300 students were enrolled in primary (grade 1–6), junior high (grade 7–9) and senior high (grade 10–12) schools, with an average of 40 students per class [27] in Nanjing. The eligible study participants were graders 4–6 (primary schools), 7–9 (junior high schools) and 10–12 (senior high schools) chosen from all the three types of schools within all 12 districts of Nanjing.

The approaches used to estimate sample size and select participants for BEACH-Children study were reported elsewhere [25, 28]. In brief, considering a multi-stage sampling method employed and the nature of cross-sectional study design, the sample size was calculated separately for testing each of the three hypothesis assumed in our BEACH-Children study: (1) 3902 participants were sufficient for examining the link between built environment and obesity; (2) 2588 for identifying the association of lifestyle and behaviors with HRQoL; and (3) 1560 for investigating HL prevalence and its association with HRQoL. Thus, the largest one (N = 3900) was determined as the final sample size for our BEACH-Children study to warrant sufficient power to test all the three hypotheses. The finalized sample size (N = 3900) was much larger than that (N = 1560) estimated for investigating HL-HRQoL association among the study population, which could allow us to examine HL-HRQoL relationship not only among overall participants but also among each stratum of gender, residence area and school type with relatively sufficient sample size in the present study.

With regard to participants’ selection, a multi-stage randomly sampling approach was applied to choose participants. Firstly, random digits were used to choose one primary, junior high and senior high school from all type-corresponding schools, separately, in each district (one district, one type-specific school). Then, we contacted those chosen schools and discussed with them about the study. When agreeing to participate in the study, the schools would provide us with the numbers of class in each grade. And, subsequently, one class was randomly determined from each study grade in the selected schools (one grade, one class) using random digits too. Finally, 108 classes were randomly chosen from 36 schools within 12 districts. Thus, all students within these 108 selected classes were the eligible participants and would receive an introduction to the study before the field survey.

Written informed consents were obtained from both participating schools and students’ parents/guardians. Prior to the survey, selected schools were firstly invited to discuss the study and to sign written informed consents if they were willing to take part into the study. Then, after receiving an introduction to the study, all eligible students were asked to bring home written informed consent for their parents/guardians reviewing before the survey. Each student was told that the parents/guardians should sign the informed consent if they agreed the child to participate in the study. Each signed informed consent was submitted to class teachers by the student after his/her parents/guardians signed it. The Ethics Committee of Nanjing Municipal Center for Disease Control and Prevention of China approved the study. Personal identification was removed prior to data analysis.

### Data collection

Participants’ socio-demographic information was gathered with a questionnaire, while the data on HL and HRQoL were assessed using specific instruments. After discussion between research team and school representatives, a specified date for field survey was determined for each participating school. Then, our filed survey was conducted school by school. On the appointed survey day, the questionnaire investigation was implemented within the regular classroom. Students self-administered the questionnaire, with two research staff and the class teacher available for assistance requested by students.

### Study variables

#### Outcome variable

HRQoL was the outcome variable and assessed with the Chinese version of Child Health Utility 9D (CHU9D-CHN) [10]. CHU9D, the first preference-based generic instrument, was developed by researchers from The University of Sheffield in The United Kingdom to specifically measure HRQoL among children and adolescents for cost-effectiveness evaluation of clinical practice or/and population-based lifestyle and behavioral intervention programs [29]. CHU9D-CHN was professionally translated from its original English version and validated among primary, junior high and senior high school students in China (Cronbach’s alpha = 0.79) [10]. Moreover, the scoring approach has been also verified for CHU9D-CHN among Chinese children and adolescents [30]. The CHU9D score ranged from 0 (worst) to 1 (best) and was used as continuous variable in our analysis.

#### Independent variable

HL was the independent variable, which was measured with the Chinese Student Health Literacy Scale (CSHLS) [31]. This HL scale, including 31 question items, was specifically developed and validated for assessing health literacy among school students in China by researchers from Fudan University School of Public Health (Cronbach’s alpha = 0.74) [31]. Similar to the scales used to assess HL for adults, the questions included in CSHLS also measure the following health-related domains: knowledge, belief and attitude, behavior and lifestyle, and practical skills [31]. Importantly, all the specific questions within each domain of CSHLS were developed with particular consideration of children and adolescents’ body growth, mental and intelligence development, and the specific cultural and social context in China.

When the scale was developed and validated, a specific scoring algorithm was also developed and recommended by the researchers [31]. The question types in the scale included true/false, single-choice and multiple-choice, with “1 point” assigned to each correct answer and “0 point” to each incorrect response, and thus resulted in a total score ranging from 0 to 31 for each student [31]. The scale developers recommended at least 80% of the total score (25 + points) as the cut-off to define sufficient/adequate HL in a population study [31]. Therefore, a participant would be classified as “having adequate HL” if he/she recorded a score of at least 25 points, otherwise “not having adequate HL” [31]. In the analysis, participants were categorized into the sub-group of “having adequate HL” or “not having adequate HL”. Moreover, HL level was defined as the proportion of participants who had adequate HL in the study.

#### Covariates

Potential confounders considered in this study were participants’ socio-demographic characteristics, including their age (years, used as continuous variable), gender (boy or girl), residential area (rural, suburban or urban area) and school type (primary, junior high or senior high school). Moreover, their parents’ educational attainment (0–9, 10–12 or 13 + years of schooling completed) was also adjusted for in the analysis as a covariate.

#### Data analysis

Firstly, the normality of HRQoL distribution was tested using Kolmogorov–Smirnov method, as the sample size was as large as more than 4000 in this study. The estimate of Kolmogorov–Smirnov analysis was 0.10 (*p* < 0.001), suggesting a non-normal distribution of HRQoL score in the study. Thus, Mann–Whitney U or Kruskal–Wallis test was applied to examine the differences in HRQoL scores (mean, standard deviation (SD)) between sub-categories. Data were also analyzed using Chi-square test to compare differences in categorical measurements (percentage) between sub-groups. Then, mean differences (MD) and 95% confidence intervals (CI) were calculated using mixed-effects linear regression models to examine HL-HRQoL associations. Two regression models were introduced. The first was a univariate model, with class-level clustering effects controlled for only. The second was a multivariate model, with adjustment for age, gender, residence area, school type, parental educational attainment, and also class-level potential clustering effects. *p* < 0.05 (two-tailed test) was regarded as significant. The data were entered with EpiData 3.1 (The EpiData Association 2008, Odense, Denmark) and analyzed using SPSS version 20.0 for Windows (SPSS Inc., Chicago, IL, USA).

## Results

### Selected characteristics of participants

Totally, 4498 students were enrolled within the 108 selected classes, and all of them were our eligible participants. There were 4388 students successfully completing the study (response rate = 97.6%). No significant difference was examined between those who took and did not take part in the survey regarding either of age, gender and residence area. Table 1 showed the selected characteristics of participants in the study. The mean age for overall 4388 respondents was 13.9 (SD: 2.5) years. Among them, 36.5%, 33.2% and 30.3% were enrolled within primary, junior high and senior high schools, respectively; 50.2% and 49.8% were boys and girls, separately; and 31.0%, 46.3% and 22.7% inhabited in urban, suburban and rural areas, respectively. There were 39.0% parents having college/above level of education, and 32.2% having senior high school level of education.

**Table 1:** Selected socio-demographic characteristic of participants by school type in this study

	Overall participants (N = 4388)	School type			*χ*^2^ value^a^	*P* value^a^
		Primary school (n = 1 602)	Junior high school (n = 1 455)	Senior high school (n = 1 331)		
Age						
Mean (SD^b^)	13.9 (2.5)	11.2 (0.9)	14.1 (0.9)	16.9 (0.9)	–	–
Gender (%)						
Boys	50.2	53.9	50.9	45.1	23.14	< 0.001
Girls	49.8	46.1	49.1	54.9		
Residence area (%)						
Rural	46.3	44.5	46.5	48.1	6.1	0.192
Suburbal	22.7	24.5	22.2	21.1		
Urban	31	31	31.3	30.8		
The highest educational level of parents (%)						
0–9 schooling years completed	28.8	25.8	35.4	25.2	149.51	< 0.001
10–12 schooling years completed	32.2	27	37.9	32.3		
13 + schooling years completed	39	47.2	26.7	42.4		

^a^Chi-square test used to compare difference between groups

^b^Standard deviation

### Health literacy and health-related quality of life among participants

Table 2 presented health literacy level and HRQoL scores among participants in the study. The proportion of participants who had adequate HL were 85.8% (95% CI = 84.7%, 86.8%). This figure was significantly higher in girls (89.1%, 95% CI = 87.7, 90.4) than boys (82.5%, 95% CI = 80.8, 84.1). Moreover, the proportion of adequate HL among participants significantly increased across sub-groups of participants from primary to senior high school, from rural to urban areas, and from lower to higher parent’s educational attainment, respectively.

**Table 2:** Distribution of adequate HL and Mean utility scores of HRQoL of participants in this study

	Number of participants	Having adequate HL^a^		*χ*^2^ value^b^	*P *value^b^	CHU9D score		*t/F* value^c^	*P *value^c^
		n	% (95% CI)			Mean	SD		
Overall	4 388	3 764	85.8 (84.7, 86.8)	–	–	0.78	0.17	–	–
Gender (%)									
Boys	2 204	1 818	82.5 (80.8, 84.1)	39.37	< 0.001	0.79	0.18	2.39	0.017
Girls	2 184	1 946	89.1 (87.7, 90.4)			0.78	0.17		
School type									
Primary school	1 602	1 182	73.8 (71.6, 75.9)	310.89	< 0.001	0.84	0.16	159.46	< 0.001
Junior high school	1 455	1 315	90.4 (88.7, 91.8)			0.78	0.16		
Senior high school	1 331	1 267	95.2 (93.9, 96.3)			0.73	0.18		
Residence area (%)									
Rural	2 030	1 779	87.6 (86.1, 89.0)	11.75	0.003	0.79	0.17	0.889	0.411
Suburban	997	848	85.1 (82.7, 87.2)			0.78	0.18		
Urban	1 361	1 137	83.5 (81.4, 85.4)			0.78	0.18		
The highest educational level of parents (%)									
0–9 schooling years completed	1 264	1 029	81.4 (79.1, 83.5)	34.86	< 0.001	0.76	0.17	12.94	< 0.001
10–12 schooling years completed	1 415	1 213	85.7 (83.7, 87.5)			0.78	0.16		
13 + schooling years completed	1 709	1 522	89.1 (87.5, 90.5)			0.8	0.18		

^a^A participant who had adequate HL referring to he/she was measured with a score of at least 25 scores points (80% of the total score)

^b^Chi-square test was applied to examine between-groups difference for categorical variables

^c^t or variance test was used to examine between-groups difference for continuous variables

The mean score of HRQoL was 0.78 (SD: 0.17) among overall participants. Girls (0.78 ± 0.17) reported significantly lower HRQoL score relative to boys (0.79 ± 0.18). Interestingly, the score for study students elevated across categories of participants from lower to higher parent’s educational level, but decreased across strata from primary to senior high schools, and from rural to urban areas, respectively.

### Relationship between HL and HRQoL among participants

Table 3 displayed the association of HL with HRQoL among participants. The mean value of HRQoL scores was found significantly different between participants with and without adequate HL (adequate vs. inadequate HL: 0.79 ± 0.17 *vs.* 0.74 ± 0.20, *P* < 0.001). After adjustment for potential confounders and class-level clustering effects, participants with adequate HL reported significantly higher HRQoL scores (MD = 0.08; 95% CI = 0.06, 0.11) than their counterparts without adequate HL. Furthermore, similar finding was observed within each stratum of participants by gender, school type and residence area.

**Table 3:** Association of health literacy with HRQoL score based on mixed-effects linear regression analysis in the study

Having or not having Adequate HL^a^		CHU9D score^b^		Model 1^c^		Model 2^d^	
		N	Mean	MD (SE)^e^	95% CI^f^	MD (SE)^e^	95% CI^f^
Overall	Not having	624	0.74	ref		ref	
	Having	3764	0.79	0.07 (0.02)	0.03, 0.10	0.08(0.01)	0.06, 0.11
Gender							
Boys	Not having	386	0.74	ref		ref	
	Having	1 818	0.8	0.07 (0.03)	0.02, 0.13	0.09(0.01)	0.06, 0.12
Girls	Not having	238	0.74	ref		ref	
	Having	1 946	0.78	0.06 (0.04)	− 0.03,0.15	0.08(0.02)	0.05, 0.11
School type							
Primary school	Not having	420	0.78	ref		ref	
	Having	1 182	0.86	0.06 (0.09)	− 0.19,0.31	0.07(0.02)	0.04, 0.10
Junior high school	Not having	140	0.69	ref		ref	
	Having	1 315	0.78	0.11 (0.04)	0.02,0.20	0.09(0.02)	0.04, 0.13
Senior high school	Not having	64	0.63	ref		ref	
	Having	1 267	0.73	0.10 (0.05)	− 0.01,0.21	0.10(0.03)	0.04, 0.16
Residence area							
Rural	Not having	251	0.72	ref		ref	
	Having	1 779	0.79	0.07 (0.02)	0.02,0.11	0.08(0.02)	0.05, 0.12
Suburban	Not having	149	0.76	ref		ref	
	Having	848	0.78	0.06 (0.03)	− 0.01,0.12	0.07(0.02)	0.03, 0.12
Urban	Not having	224	0.76	ref		ref	
	Having	1 137	0.78	0.07 (0.03)	0.002, 0.14	0.09(0.02)	0.04, 0.14

^a^A participant who had adequate HL referring to he/she was measured with a score of at least 25 scores points (80% of the total score)

^b^CHU9D utility score was treated as continuous variable in the analysis

^c^Model 1 was a univariate mixed-effects linear regression model with HL as the main effect and adjustment for class-level clustering effects controlled for only

^d^Model 2 was a multivariate mixed-effects linear regression model, with adjustment for age, gender, residence area, school type, parental educational attainment and class-level clustering effects

^e^MD referred to Mean Difference between groups, while SE was standard error

^f^CI meant confidence interval

## Disscussion

In this population-based study, we aimed to investigate the relationship between HL and HRQoL among school-aged children and adolescents in China. It was examined that HL level was positively associated with HRQoL score among either overall participants or each sub-group of gender, school type and residence area, with consideration of participants’ socio-demographic characteristics and their parents’ educational level. This may imply that such a significantly positive association of HL with HRQoL is likely to exist independently among primary, junior high and senior high school students in China.

The findings among students from our study were consistent with that have been documented in adults [21]. Moreover, although very few studies regarding HL-HRQoL association were available from children and adolescents worldwide, such a positive HL-HRQoL relationship observed in our study was also in line with that reported among children and adolescents in similar studies from Virginia of USA [22], Belo Horizonte city of Brazil [23] and Chongqing city of China [24]. However, the age-groups of participants were different between our study and the three mentioned above [22–24]. Participants were students aged 11–13 years in the Virginia study [22], 15–19 years in the Belo Horizonte study [23] and 12–15 years in the Chongqing study [24], while participant’s age ranged widely from 9 to 17 years in our study. Furthermore, the instruments used to assess either HL or HRQoL were different, but the consistent findings were observed in the three studies and ours [22–24]. Therefore, it may suggest that a positive HL-HRQoL relationship may solidly hold for children and adolescents within different age-groups and under different social and cultural contexts.

There were at least two mechanisms to explain the HL-HRQoL association identified in this study. The first was based on the Knowledge, Attitude and Practices theory (KAP model), one of the widely used theories regarding recognition and behavior change [32]. According to the KAP model, a person’s practices (behaviors) are determined by his/her knowledge and attitudes toward the behaviors. So, if a person acquired relevant knowledge, and had his/her attitudes/beliefs generated, he/she would form specific practices and then his/her behaviors could be effectively changed and subsequently maintained [32]. Health literacy is a comprehensive concept of capacity about health-related knowledge, belief, skills and behaviors [14, 15], therefore from the perspective of KAP theory individuals with higher HL score may reasonably have more health-related knowledge, and then hold more healthy belief, and consequently take more favorable skills and behaviors to maintain their general health state relative to their counterparts with lower HL score. Meanwhile, HRQoL is a subjective and broad perception regarding physical and mental health conditions [1–3], which implies that people with better general physical or/and mental health state may have better perception of HRQoL. Thus, it can partly explain the positive HL-HRQoL relationship observed in this study by that students with adequate HL may have better overall health state and then result in better perception of HRQoL than those without adequate HL.

The other mechanism behind the positive HL-HRQoL association may be the mediation of lifestyle and behavior, as it has been well documented that some lifestyle and behaviors, e.g., insufficient physical activity, prolonged screen time, fast-food and soft-drink consumption, are in negative relation to HRQoL in children and adolescents [4–10]. And, this was also supported by findings from the data of our study [11]. Meanwhile, students with lower HL score may tend to engage in these risky lifestyle and behaviors [33]. So it can be, at least in part, to explain the HL-HRQoL association identified in our study by that those individuals with lower HL might hold some unhealthy lifestyle and behaviors and thus consequently perceived poorer HRQoL.

Childhood and adolescence are critical for an individual on either body growth or mental and intelligence development in that, compared to adults, children and adolescents: (1) have less well-developed cognitive and decision-making ability, (2) are more easily to be influenced regarding life attitudes/beliefs by their care-givers and peers, and (3) may experience different lifestyle and behavior patterns, physical and/or mental health conditions [34, 35]. Meanwhile, of the HL domains, health-related knowledge was the core and the key element, and should be paid particular attention to in population-based HL promotion campaigns [36]. Thus, considering the development attributes of childhood and the key role of health-related knowledge involved in HL, it is really important to educate children and adolescents to correctly obtain and adequately utilize sufficient health-related knowledge within well-developed school-based health promotion programs, as it may have optimal effectiveness of input–output for students’ HL promotion.

This study had several strengths. First, the instruments used to assess HRQoL and HL were specifically developed and validated for children and adolescents, warranting reliable and effective instruments used. Second, the randomly chosen participants were with a wide range of age and from urban, suburban and rural areas in a typical mega-city of China, suggesting a representative sample of overall school students recruited in the study. Third, 97.6% of 4498 eligible participants from primary, junior and senior high schools successfully completed the survey, showing a very high response rate obtained. Last, consistent findings were observed among overall and all the strata of participants by age, gender and residence area, implying the HL-HRQoL association was solid with generalizability among children and adolescents in China.

In addition to strengths mentioned above, some limitations should also be addressed. Firstly, the nature of cross-sectional study design could not warrant a causality of the HL-HRQoL relationship. Secondly, some potential confounders might not be adjusted for in the analysis, although we have put into consideration of students’ demographic characteristics and clustering-effects at class level. Finally, based on the present HL instrument, the proportion of sufficient HL was very high among participants in the study. This might imply a big challenge that it would be not easy to further improve population-level HL and thus the output-input effectiveness of HL intervention campaigns among students would be not as high as expected in China.

In future, well-designed longitudinal observational studies are encouraged to further understand the HL-HRQoL association among large scale general population of children and adolescents, while school-based health education programs are needed to assess the effectiveness of HL intervention on HRQoL promotion for students.

## Conclusisons

In this population-based study, health literacy was examined to be positively associated with health-related quality of life among overall and each specific stratum of students by age, gender and residence area in regional China. The findings from this study have important implications from public health perspectives that HRQoL may be improved through school-based health literacy intervention among children and adolescents in China.

## Data Availability

Data are available upon request to corresponding authors.
